# The Cellular Senescence Factor Extracellular HMGB1 Directly Inhibits Oligodendrocyte Progenitor Cell Differentiation and Impairs CNS Remyelination

**DOI:** 10.3389/fncel.2022.833186

**Published:** 2022-04-28

**Authors:** Megan E. Rouillard, Jingwen Hu, Pearl A. Sutter, Hee Won Kim, Jeffrey K. Huang, Stephen J. Crocker

**Affiliations:** ^1^Department of Neuroscience, University of Connecticut School of Medicine, Farmington, CT, United States; ^2^Department of Biology and Center for Cell Reprogramming, Georgetown University, Washington, DC, United States

**Keywords:** cellular senescence, myelin, multiple sclerosis, lysolecithin (LPC), microglia, TLR2, spinal cord

## Abstract

HMGB1 is a highly conserved, ubiquitous protein in eukaryotic cells. HMGB1 is normally localized to the nucleus, where it acts as a chromatin associated non-histone binding protein. In contrast, extracellular HMGB1 is an alarmin released by stressed cells to act as a danger associated molecular pattern (DAMP). We have recently determined that progenitor cells from multiple sclerosis patients exhibit a cellular senescent phenotype and release extracellular HMGB1 which directly impaired the maturation of oligodendrocyte progenitor cells (OPCs) to myelinating oligodendrocytes (OLs). Herein, we report that administration of recombinant HMGB1 into the spinal cord at the time of lysolecithin administration resulted in arrest of OPC differentiation *in vivo*, and a profound impairment of remyelination. To define the receptor by which extracellular HMGB1 mediates its inhibitory influence on OPCs to impair OL differentiation, we tested selective inhibitors against the four primary receptors known to mediate the effects of HMGB1, the toll-like receptors (TLRs)-2, -4, -9 or the receptor for advanced glycation end-products (RAGE). We found that inhibition of neither TLR9 nor RAGE increased OL differentiation in the presence of HMGB1, while inhibition of TLR4 resulted in partial restoration of OL differentiation and inhibiting TLR2 fully restored differentiation of OLs in the presence of HMGB1. Analysis of transcriptomic data (RNAseq) from OPCs identified an overrepresentation of NFκB regulated genes in OPCs when in the presence of HMGB1. We found that application of HMGB1 to OPCs in culture resulted in a rapid and concentration dependent shift in NFκB nuclear translocation which was also attenuated with coincident TLR2 inhibition. These data provide new information on how extracellular HMGB1 directly affects the differentiation potential of OPCs. Recent and past evidence for elevated HMGB1 released from senescent progenitor cells within demyelinated lesions in the MS brain suggests that a greater understanding of how this molecule acts on OPCs may unfetter the endogenous remyelination potential in MS.

## Introduction

Multiple sclerosis (MS) is a debilitating autoimmune disease affecting myelination of the central nervous system (CNS). Worsening disability among MS patients with progressive forms of disease is attributable to chronic loss of oligodendrocytes (OLs) and myelin, as well as the failure of oligodendrocyte progenitor cells (OPCs) to differentiate into mature OLs to replace lost myelin (Nicaise et al., 2019). This differentiation failure occurs even as OPCs populate the demyelinated lesions in the MS brain, indicating the presence of anti-differentiation factors or the loss of pro-differentiation factors (Wolswijk, 1998, 2002; Chang et al., 2002). It is hypothesized that manipulating these factors may offer a mechanism to halt disease progression and restore neurological function by promoting myelination in the brain and spinal cord (Brück et al., 2013; Faissner and Gold, 2019).

Neural progenitor cells (NPCs) are an early progenitor cell type with the capability to differentiate into neurons, astrocytes, or OLs, depending on the environmental cues they encounter (Chang et al., 2002; Gudi et al., 2014). Previous work in our lab determined that induced pluripotent stem cells (iPSCs) obtained from primary progressive MS (PPMS) patients and subsequently differentiated to NPCs were deficient in supporting myelin regeneration both *in vitro* and *in vivo* when compared to iPS-derived NPCs from age matched controls (Nicaise et al., 2017). It was initially reported, and recently independently confirmed by others (Mutukula et al., 2021), that NPCs from PPMS samples *in vitro* exhibit a senescent phenotype, and validated the finding that Sox2+ progenitor cells in progressive MS brain tissues expressed elevated levels of cellular p16, p21, p53, and other markers (Nicaise et al., 2019). This finding indicated that the conditioned media (CM) from iPS-derived NPCs from PPMS patients represents a disease-associated senescent secretory phenotype. A proteomic analysis of the NPC secretome was conducted and PPMS NPCs were found to secrete high levels of high mobility group box 1 (HMGB1) that control NPCs did not secrete high levels of HMGB1 (Nicaise et al., 2019). HMGB1 was also found to colocalize to Sox2+ cells within the white matter lesions in autopsy pathology. Moreover, when a function blocking antibody against HMGB1 was applied to the CM derived from PPMS patient iPS-derived NPCs, maturation of OPCs increased significantly. This indicates an important role for HMGB1 in the influence of senescence on limiting OL-mediated remyelination (Nicaise et al., 2019).

HMGB1, also known as amphoterin, is a highly conserved, ubiquitous protein found in nearly all eukaryotic cells. It is highly conserved between rodents and humans, with the sequence homology exceeding 98% (Ellerman et al., 2007). HMGB1 is recognized as the prototypical alarmin, or danger/damage associated molecular pattern (DAMP). This class of proteins have functions in the unstressed cell but are actively secreted following stress or damage, provoking an innate immune response (Robinson et al., 2013). Under normal circumstances, HMGB1 remains in the cell, acting in the nucleus as the most abundant chromatin associated non-histone binding protein, stabilizing nucleosomes and regulating the transcription of many genes (O’Connor et al., 2003; Ellerman et al., 2007). In contrast, extracellular HMGB1 has been found to be a mediator of both sterile inflammation and infection associated responses (Yang and Tracey, 2010), which can induce proinflammatory cytokine release from cells. HMGB1 has been implicated in a variety of inflammatory conditions including sepsis, inflammatory bowel disease, pancreatitis, acute lung injury, rheumatoid arthritis, hemorrhagic shock lacking infection, traumatic brain injury, stroke, depression, and ischemia-reperfusion injury (Wang et al., 1999; O’Connor et al., 2003; Yang and Tracey, 2010; Lian et al., 2017). Importantly, treatment of animal models of diseases with anti-HMGB1 antibodies has been shown to be effective in reducing the impact of these conditions (O’Connor et al., 2003; Robinson et al., 2013). While HMGB1 is considered a cytokine when released from immune cells, our data indicates that HMGB1 acts on OPCs, suggesting a direct receptor-mediated function. Extracellular HMGB1 has several identified receptors, including the toll-like receptors (TLRs) TLR2, TLR4, and TLR9 and the receptor for advanced glycation end products (RAGE; Hori et al., 1995; Hoarau et al., 2011). TLRs belong to a family of pattern recognition receptors capable of recognizing a large range of pathogen or danger associated molecular patterns (PAMPs or DAMPs), both of which can trigger an immune response. HMGB1 has been found to promote production of inflammatory factors by binding to TLRs (Andersson et al., 2000) and promote inflammation by binding to RAGE (Watanabe and Son, 2021). It is currently unknown which of these receptors and subsequent pathways HMGB1 acts on in OPCs to prevent maturation.

Herein we report that the HMGB1 suppresses OPC maturation by a TLR2mediated mechanism and co-application of recombinant HMGB1 to spinal cord lysolecithin (LPC) lesions significantly impairs remyelination. Taken together, these data demonstrate a potentially important extracellular role for HMGB1 as a factor involved in impaired remyelination in the multiple sclerosis brain.

## Materials and Methods

### Animals

Rat pups used in this study were the offspring of WT Sprague Dawley rats purchased from Charles River. C57BL/6J wild-type mice of both sexes were purchased from the Jackson Laboratory. Mice were accommodated for a week after receiving and were maintained on a 12-h light/12-h dark cycle with food and water *ad libitum* throughout the experiments. All experiments were performed in accordance with protocols approved by either the University of Connecticut School of Medicine or Georgetown University Institutional Animal Care and Use Committees. Procedures were also conducted following the guidelines set forth by the National Research Council of the National Academies *Guide for the Care and Use of Laboratory Animals*.

### Lysolecithin Lesions

Focal demyelinated lesions were induced by injecting 1 μl of 1.0% LPC (Sigma-Aldrich, MA, USA) in PBS into the spinal cord ventral funiculus of 11-week-old mice as previously described (Psachoulia et al., 2016). For the treatment group, 1 μl of 1 mg/ml recombinant human HMGB1 protein (Abcam, Cambridge, UK) in water was co-injected along with 1.0% LPC into the ventral spinal cord. For the control group, 1 μl of 1 mg/ml recombinant human HMGB1 protein (Abcam, Cambridge, UK) in water was co-injected with the vehicle, sterile phosphate buffered saline (PBS).

### Sample Processing

Mice were perfused intracardially with 4% fresh paraformaldehyde (PFA, Sigma-Aldrich, MA, USA) at 10 days post lesion. Spinal cords were dissected and post-fixed with 4% PFA before being cryoprotected in 30% (w/v) sucrose solution (Sigma-Aldrich, MA, USA) in PBS at 4°C overnight. The tissue was then frozen in O.C.T. (Fisher Scientific, MA, USA) on dry ice and stored at −80°C. Spinal cord demyelinated lesions were serially collected on SuperFrost^®^Plus slides (VWR International, PA, USA) using a cryostat (CM1900; Leica, Wetzlar, Germany). Sections were cut to 12 μm, and were dried for 30 min at room temperature before storing at −80°C.

### Immunohistochemistry

Spinal cord demyelinated sections were dried for 1 h at room temperature before performing immunohistochemistry as previously described (Baydyuk et al., 2019). Antigen retrieval was performed for Olig2 staining. Sections were washed with 0.05% Tween 20 in TBS (Sigma-Aldrich, MA, USA) followed by TBS to remove O.C.T. on the slides. For permeabilization, sections were washed with 1% Triton X-100 (Sigma-Aldrich, MA, USA) in TBS for 15 min. The samples were then incubated in blocking solution (5% normal goat serum, 5% donkey serum, and 0.25% Triton X in TBS) for 1 h at room temperature, followed by mouse-on-mouse IgG blocking solution according to the manufacturer’s instructions (Vector Laboratories, CA, USA) when using mouse primary antibodies. Subsequently, the sections were incubated at 4°C overnight in primary antibodies diluted in blocking solution (1:300 rabbit anti-Olig2, EMD Millipore; clone CC-1, EMD Millipore; 1:100 mouse anti-Nkx2.2, Developmental Studies Hybridoma Bank; 1:400 rabbit anti-Iba1, Wako Chemicals USA, Inc. ANTI; 1:100 mouse Anti-iNOS/NOS Type II, BD Biosciences; 1:500 rat anti-myelin basic protein, Millipore). On the following day, slides were washed with 0.05% Tween 20 in TBS followed by TBS, then incubated in secondary antibody solution (1:1,000 Cy3-conjugated AffiniPure donkey anti-rabbit IgG (H + L), Jackson ImmunoResearch; 1:500 Alexa Fluor 488 goat anti-mouse IgG (H + L), Thermo Fisher; 1:500 Alexa Flour 594 donkey anti-rat IgG (H + L), Thermo Fisher, 1:1,000 Hoechst 33342, Thermo Fisher, MA, USA) for 1 h at room temperature. Sections were then washed again with 0.05% Tween 20 in TBS followed by TBS to remove excessive secondary antibodies before mounting. To avoid variable staining quality among different batches, slides from all groups were stained on the same day for each type of staining. Images were taken with Zeiss LSM800 confocal microscope (Zeiss, Oberkochen, Germany). To avoid image differences due to laser stability, images from all groups were taken on the same day with the same confocal settings for each type of staining. DAPI intensity was referred to ensure the staining quality was consistent throughout the slides. For Olig2, CC1, Nkx2.2, Iba1, and iNOS staining, cells were counted in the lesioned area defined by Hoechst staining and then normalized to the lesion area (mm^2^) of each section. Spinal meninges are excluded from the counts. For myelin basic protein (MBP) staining, the percentage of MBP+ area coverage in lesions was determined by Image J. The results of 2–6 spinal cord sections from each mouse were used for quantification, 3–5 mice were examined in each group.

### Isolation of Rat OPCs and Treatment With HMGB1 and Inhibitors

OPCs were isolated from the cerebral cortices of neonatal rat pups (postnatal day 0–3) as previously described (Moore et al., 2011). Cells were plated on poly-l-ornithine (Sigma-Aldrich, MA, USA) coated glass coverslips, as previously described (Moore et al., 2011). OPCs were treated with 500 μl of differentiation media [neurobasal media; Gibco, 2% B27, 1% L-glutamine (2 mM) and 0.3% T3 (10 ng/ml)] containing recombinant human HMGB1 (0.5 μg/ml; Abcam, Cambridge, UK) or recombinant human HMGB1 (0.5 μg/ml) and the indicated concentration of each inhibitor. All tests were performed with HMGB1 and the inhibitor in differentiation media, with differentiation media alone serving as the control. Concentrations were determined from the manufacturer provided IC50. The following drugs were used: CU CPT22 (TLR2/1 specific inhibitor; 0.5 and 1 μM; Tocris), C34 (TLR4 specific inhibitor, 5 and 10 μM; Tocris), Hydroxychloroquine sulfate (HQS; TLR9 specific inhibitor, 1 and 2 μM; Tocris, Bristol, UK), and FPS-ZM1 (RAGE specific inhibitor; 3 and 6 μM; Tocris, Bristol, UK). All drugs were reconstituted in accordance with the manufacturer instructions. Coverslips were treated for 72 h.

### Immunocytochemistry

Following treatment, coverslips were gently washed with PBS before fixation with 4% PFA. Following fixation, cells were permeabilized using 5% goat serum (Thermo Fisher Scientific, MA, USA) and 0.01% Tween20 (Sigma-Aldrich, MA, USA). Cells were stained using 4’6-diamidino-2-phenylindole (DAPI) to identify nuclei, as well as the indicated primary antibodies, including MBP (1:500; Abcam, Cambridge, UK), Olig2 (1:500; Abcam, Cambridge, UK), and NFκB (1:200; Thermo Fisher Scientific, MA, USA). Conjugated secondary antisera directed against the species of the primary were used according to manufacturer instructions (1:1,000, Abcam, Cambridge, UK). Coverslips were then affixed to slides (Denville Ultraclear, MA, USA) with fluromount-G (Invitrogen, MA, USA) and imaged (Olympus 1X71, CellSens Software; Olympus, MA, USA). Five fields of view at 20× magnification using identical image capture settings were assessed by an experimenter blinded to treatments. To analyze the amount of OPC differentiation, Olig2+ cells and MBP+ cells were counted and the percent MBP+ cells was calculated. Olig2+ was also used to distinguish astrocytes in the cultures to refine the cell counts to only MBP+/Olig2+ OL-lineage cells. Data are presented as the percentage of MBP+/Olig2+ cells relative to the control, differentiation media only, condition set as 100%.

### Gene Ontology (GO) Enrichment Analysis of NFκB Target Genes

Gene expression analyses using our previously reported RNAseq datasets were used to identify HMGB1-specific transcriptional changes across OPCs under defined treatment conditions, as previously described (Nicaise et al., 2019). Significantly up or downregulated genes in PPMS vs. PPMS+αHMGB1 were generated (*p* < 0.05, LogFoldChange > ±1). Differentially expressed genes (DEGs) were then cross-referenced with a list of known NFκB genes (Rouillard et al., 2016) to determine which DEGs were also known to be NFκB regulated. These filtered gene lists were then analyzed using the PANTHER Pathway analysis tool^1^ linked through the Gene Ontology Consortium’s online database^2^ (Mi et al., 2013). Gene names were copied into the PANTHER Pathway analysis tool, where we selected organism (*Rattus norvegicus)*, specific enrichment analysis (*Biological* or *Reactome*), statistical test type and correction (*Fisher’s Exact* with *calculation of FDR)*. Significant results were identified by filtering by *false discovery rate (FDR < 0.05)* and *P*-value (*p* value < 0.01). We reported significant findings using a 3D representation showing the *P*-value and fold enrichment for each significantly enriched pathway.

### Statistical Analysis

Statistics for ICC were performed using GraphPad Prism 8 (La Jolla, CA, USA). Statistics for IHC were performed using GraphPad Prism 9 (La Jolla, CA, USA). Data are represented as mean ± SEM. Significance was determined using one-way ANOVA. Statistical significance is reported as not significant (n.s.) *P* > 0.05, *P ≤ 0.05, **P ≤ 0.01, ***P ≤ 0.001, ****P ≤ 0.0001.

## Results

### HMGB1 Inhibits Oligodendrocyte Differentiation in a LPC Model of Demyelination

**Figure 1:**
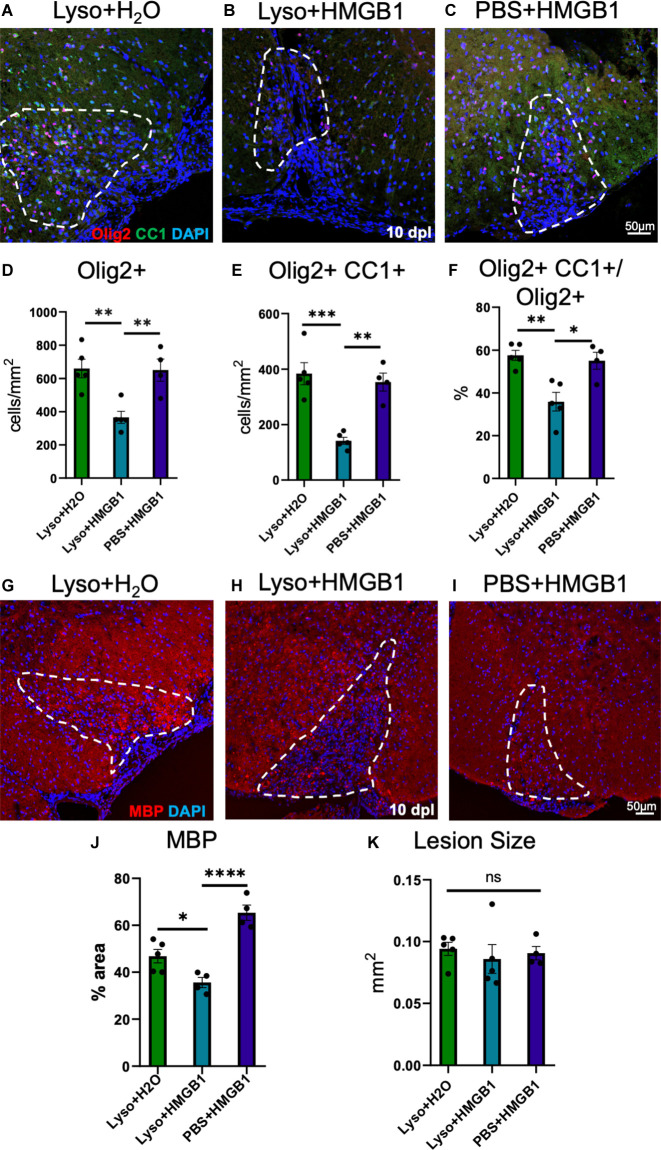
HMGB1 reduced evidence for myelination after LPC-induced demyelination. **(A)** A representative IHC image of mouse spinal cord injected with 1 μl LPC (1.0%) and water control 10 days post lesion (dpl) stained with Olig2, CC1, and DAPI. **(B)** A representative IHC image of mouse spinal cord co-injected with 1 μl LPC (1.0%) and 1 μl rhHMGB1 (1 mg/ml in water) at 10 dpl stained with Olig2, CC1, and DAPI. **(C)** A representative IHC image of mouse spinal cord injected with PBS and 1 μl rhHMGB1 (1 mg/ml in water) at 10 days post injection stained with Olig2, CC1, and DAPI. **(D)** Quantification of the number of Olig2+ cells. **(E)** Quantification of the Olig2+ and CC1+ cells. **(F)** Percent of Olig2+ cells that were also CC1+. **(G)** Representative image of mouse spinal cord injected with 1 μl LPC (1.0%) and water control 10 dpl stained for myelin basic protein (MBP) and DAPI. **(H)** A representative IHC image of mouse spinal cord co-injected with LPC and rhHMGB1 at 10 dpl stained for myelin basic protein (MBP) and DAPI.** (I)** A representative IHC image of mouse spinal cord injected with PBS and 1 μl rhHMGB1 (1 mg/ml in water) at 10 days post injection stained for myelin basic protein (MBP) and DAPI. **(J)** Percent of the lesion area that was MBP+. **(K)** Quantification of the area of the lesion in mm^2^. White dashed lines indicate lesion/injection site. Two to six spinal cord sections from each mouse were used for quantification, 3–5 mice per condition. ****P ≤ 0.0001; ***P ≤ 0.001; **P ≤ 0.01; and *P ≤ 0.05. ns when *P* > 0.05. Scale bar = 50 μm.

**Figure 2:**
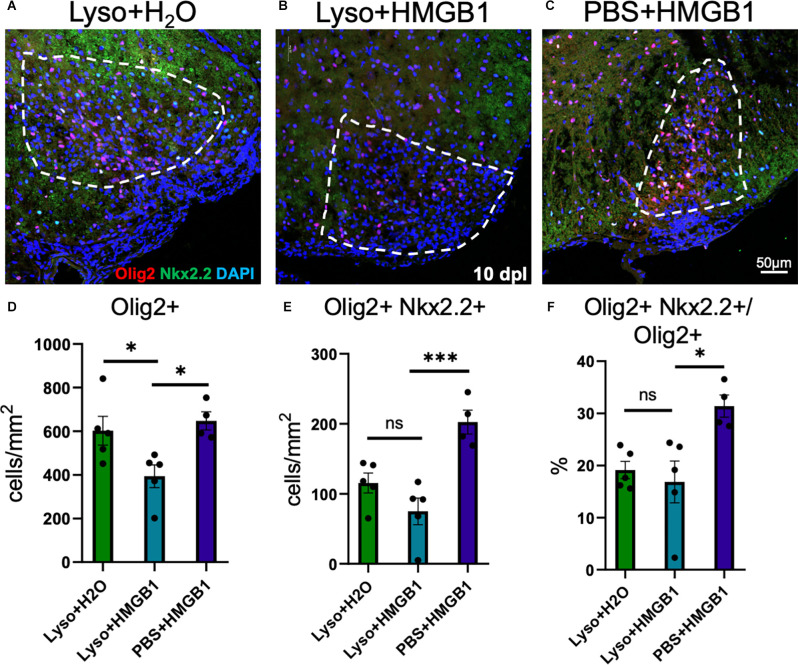
HMGB1 delayed maturation of oligodendrocytes *in vivo*. **(A)** A representative IHC image of mouse spinal cord injected with 1 μl LPC (1.0%) and water control 10 dpl stained for Olig2, Nkx2.2, and DAPI. **(B)** A representative IHC image of mouse spinal cord co-injected with 1 μl LPC (1.0%) and 1 μl rhHMGB1 (1 mg/ml in water) at 10 dpl stained for Olig2, Nkx2.2, and DAPI. **(C)** A representative IHC image of mouse spinal cord injected with 1 μl rhHMGB1 (1 mg/ml in water) 10 days post injection stained for Olig2, Nkx2.2, and DAPI. **(D**–**F)** Quantification of lesions, including Olig2+ cells, Olig2+, and Nkx2.2+ cells and the percent of Olig2+ cells that were also Nkx2.2+. White lines indicate lesion/injection site. Two to six spinal cord sections from each mouse were used for quantification, 3–5 mice per condition. ***P ≤ 0.001 and *P ≤ 0.05. ns when *P* > 0.05. Scale bar = 50 μm.

**Figure 3:**
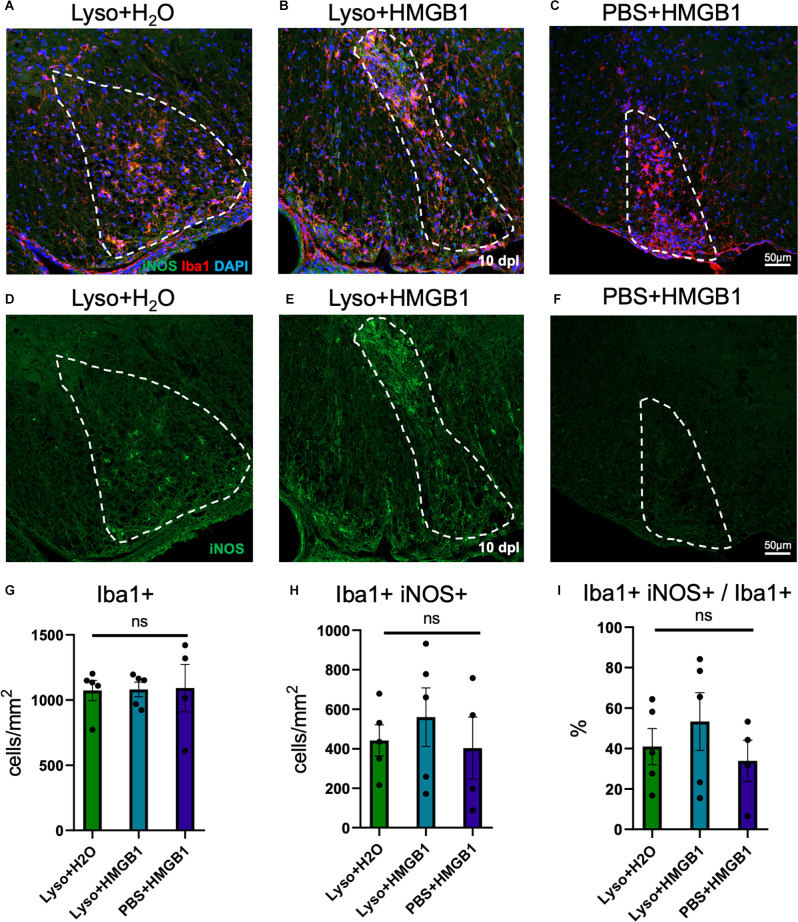
HMGB1 treatment did not increase inflammation within demyelinated lesions. **(A)** A representative IHC image of mouse spinal cord injected with 1 μl LPC (1.0%) and water control 10 dpl stained with iNOS, Iba1, and DAPI. **(B)** A representative IHC image of mouse spinal cord co-injected with 1 μl LPC (1.0%) and 1 μl rhHMGB1 (1 mg/ml in water) at 10 dpl stained with iNOS, Iba1, and DAPI. **(C)** A representative IHC image of mouse spinal cord injected with 1 μl rhHMGB1 (1 mg/ml in water) 10 days post injection stained with iNOS, Iba1, and DAPI. **(D**–**F)** Single channel images of iNOS immunostaining of samples represented in panels **(A**–**C)**. Lesion areas are represented by dashed white lines. **(G)** Quantification of Iba1+ cells. **(H)** Quantification of Iba1+ and iNOS+ cells. **(I)** Percent of Iba1+ cells that were also iNOS + . White dashed lines indicate lesion/injection site. Two to six spinal cord sections from each mouse were used for quantification, 3–5 mice per condition. ns when *P* > 0.05. Scale bar = 50 μm.

HMGB1 has been shown to be detrimental to OPC differentiation *in vitro* (Nicaise et al., 2019) and has been found to be significantly elevated in MS patient plasma (Bucova et al., 2020). Higher levels of HMGB1 have also been linked with active lesions as well as an association between HMGB1 levels and disability (Bucova et al., 2020). To determine whether HMGB1 introduced into an active lesion *in vivo* would impact remyelination, we co-injected recombinant human HMGB1 with LPC into mouse spinal cords and assessed lesion volume 10 days later (data post lesion, dpl). Animals that had been co-injected with LPC and HMGB1 were found to have significantly fewer mature Olig2+/CC1+ OLs compared to those co-injected with LPC and vehicle (H_2_O; Figure 1). The lack of mature oligodendrocytes in the lesion environment was not due to insufficient numbers of early stage oligodendrocyte lineage cells because we also found that the numbers of Olig2+/Nkx2.2+ cells, which identifies activated OPCs during remyelination (Fancy et al., 2004), were present at the same levels in the LPC lesions whether co-injected with HMGB1 or vehicle (PBS; Figure 2). These findings indicated that HMGB1 effectively impaired the differentiation of oligodendrocytes when introduced into the LPC lesion environment. To determine if administration of HMGB1 into the LPC lesion affected the degree of remyelination, as indicated by production of myelin basic protein (MBP+), we also assessed the amount of MBP+ immunostaining in each treatment condition and found reduced MBP+ levels in mice that had been LPC lesioned and also treated with HMGB1 (Figure 1). To determine if the reduced numbers of mature OLs (CC1+) and myelin (MBP+) in LPC/HMGB1-treated animals was attributed to an overall loss of oligodendrocyte lineage cells in the lesion environment, we then analyzed Olig2+/Nkx2.2+ OPCs in the lesions of each treatment group. This analysis determined that the HMGB1 did not reduce the numbers of OPCs in LPC-treated animals relative to LPC-Vehicle (H_2_O) treated subjects (Figure 2). We did note that when compared with non-LPC + HMGB1 injected animals, the number of OPCs was higher than in LPC-lesioned animals which indicated that HMGB1 alone did not impact the survival of OL-lineage cells. This was consistent with previous findings that LPC kills nearly all oligodendroglial cells around the injection site, and new OPCs migrating in from outside the lesion are needed for remyelination (Baydyuk et al., 2019). To futher define and compare the state of the demyelinated lesions in each treatment group at this 10 dpl timepoint, we also examined iNOS + /Iba1+ microglia/macrophages as an indicator of active demyelination. This analysis determined that there were no significant differences in the numbers of Iba1+ activated microglia or iNOS + /Iba1+ cells in either LPC treatment group at the timepoint analyzed (Figure 3). Hence, these data indicate that a single bolus injection of HMGB1 introduced into an active lesion environment was sufficient to effectively impair the recovery and remyelination of an LPC lesion.

### TLR2/1 Inhibitor CU CPT22 Rescued Differentiation in Oligodendrocyte Progenitor Cells Treated With HMGB1

Our previous and current data indicate that HMGB1 can directly inhibit the differentiation of OPCs both *in vivo* and *in vitro*. To understand how extracellular HMGB1 influences OPCs, we examined whether any known HMGB1 receptors were involved and also examined which intracellular signaling pathway it was likely activating. Based on what is known about the action of extracellular HMGB1 in other cell types and disease models, we hypothesized that HMGB1 was likely acting through one of four receptors: TLR2, TLR4, TLR9 or RAGE (Hori et al., 1995; Park et al., 2004; Hoarau et al., 2011). To test this hypothesis, we utilized receptor-specific inhibitors to determine which receptor HMGB1 was acting through in OPCs.

**Figure 4:**
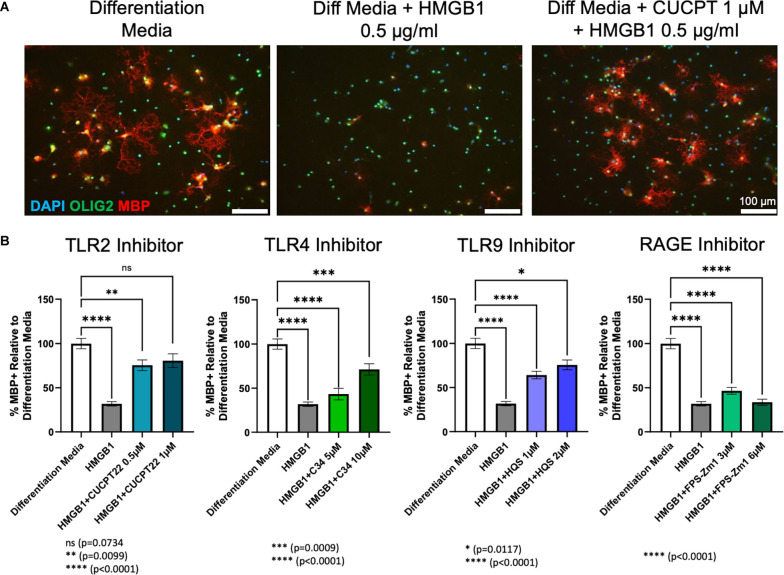
HMGB1 inhibited OPC differentiation *via* TLR2/1 receptors. **(A)** Differentiation of rat OPC cultures after 72 h of treatment with differentation media, differentiation media plus HMGB1 or differentiation media plus HMGB1 and CU CPT22. **(B)** Quantification of the amount of differentiation, as measured by the percent of MBP+ cells, normalized to differentiation media using each receptor inhibitor [Not shown: all conditions are significantly different from HMGB1 (*p* < 0.0001), except FPS-Zm1 3 μM and 6 μM (ns)]. Analyzed by one-way ANOVA, where ****P ≤ 0.0001; ***P ≤ 0.001; **P ≤ 0.01; and *P ≤ 0.05. ns when *P > 0.05*. Scale bar = 100 μm.

OPC cultures obtained from wild type (WT) Sprague Dawley rat pups (P0–P3) were treated for 72 h with each receptor inhibitor and 0.5 μg/ml HMGB1 in differentiation media. Consistent with prior research (Nicaise et al., 2019), adding HMGB1 to differentiation media significantly diminished the amount of MBP produced by oligodendroglial lineage cells when compared to differentiation media alone. This indicated a failure of OPCs to differentiate into mature, myelinating OLs in the presence of HMGB1 *in vitro*. Treatment with the inhibitors of TLR4 (C34), TLR9 (hydroxychloroquine sulfate) or RAGE (FPS-ZM1) in the presence of HMGB1 was unable to restore differentiation to control levels. However, the TLR4 and TLR9 inhibitors were able to produce significantly more differentiation than the HMGB1 only condition. In contrast, treatment of OPCs with CU CPT22 (1 μM, a TLR2/1 specific inhibitor) in the presence of HMGB1 (0.5 μg/ml) resulted in a significant increase in the percentage of mature OL lineage cells (Olig2+/ MBP+). HMGB1 and CU CPT22 co-treated cultures exhibited no significant differences in differentiation when compared to differentiation media only control conditions (Figure 4), suggesting that HMGB1-mediated inhibition of OPC differentiation is mediated, at least in part, by TLR2/1.

### HMGB1 Regulates NFκB Signaling in OPCs

**Figure 5:**
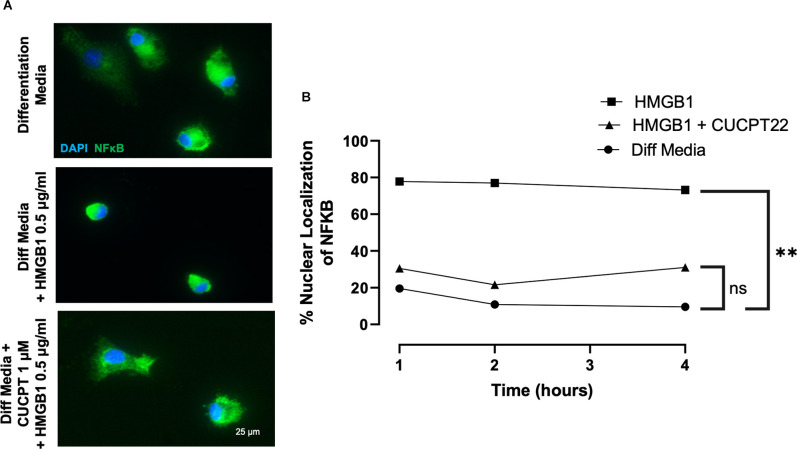
HMGB1 promoted nuclear localization of NFκB after HMGB1 treatment. **(A)** Immunocytochemical detection of NFκB in primary OPC cultures identified NFκB to be predominantly in the cytoplasm of control, differentiating cells, but was observed to be rapidly localized to the nucleus when exposed to HMGB1. Addition of the TLR2 inhibitor, CU CPT22, prevented the nuclear localization of NFκB in HMGB1-treated OL lineage cells. **(B)** Quantification of the percent of OL lineage cells with nuclear localization of NFκB over time. Analyzed by repeated measures ANOVA, where **P ≤ 0.01; and ns when *P* > 0.05. Scale bar = 20 μm.

To define how HMGB1 may regulate the transcriptional responses of OPCs, we noted that NFκB is a common downstream target of TLR2/1 signaling. Nuclear factor κB (NFκB) is a well-established downstream target of TLR2/1 and has also been found to be activated in chronic MS lesions in OLs (Bonetti et al., 1999). NFκB is a transcription factor comprised of at least five cytoplasmic proteins [RelB, c-Rel, p50, p52, and p65 (RelA)] and is inhibited by IκB, which sequesters NFκB in the cytoplasm where it is inactive. Activating NFκB requires degradation of IκB, allowing NFκB to translocate into the nucleus where the Rel subunits can bind target genes and begin transcription (Bonetti et al., 1999). To evaluate the effects of HMGB1 on the intracellular localization of NFκB in OPCs, we treated OPCs with HMGB1 and fixed at various timepoints, then immunostained for NFκB to determine if subcellular localization was influenced by HMGB1. In HMGB1-treated OPCs we found 80% of cells exhibited nuclear localization of NFκB after 1-, 2- and 4-h of treatment (Figure 5). This was in contrast to cells treated with only differentiation media, where less than 20% of the OPCs had any notable nuclear NFκB localization. Thus, the nuclear translocation of NFκB in response to HMGB1 suggests that this pathway may represent a means by which HMGB1 could influence gene transcription and OPC differentiation.

To evaluate whether regulation of TLR2/1 signaling also impacted the nuclear localization of NFκB in response to HMGB1, we examined the subcellular localization of NFκB in HMGB1 treated OPCs with and without the TLR2/1 inhibitor CU CPT22. We observed CU CPT22 and HMGB1 co-treated cells did not exhibit significant nuclear localization of NFκB and that the proportion of cells with nuclear NFκB did not differ significantly from OPCs cultured in differentiation media alone (Figure 5). These results suggest that HMGB1 activation of NFκB regulated nuclear transcription can be regulated or mediated by TLR2. Hence, identification of NFκB target genes that are transcribed in response to HGMB1 may represent future candidates for mediating the arrest of OPC differentiation by HMGB1.

**Figure 6:**
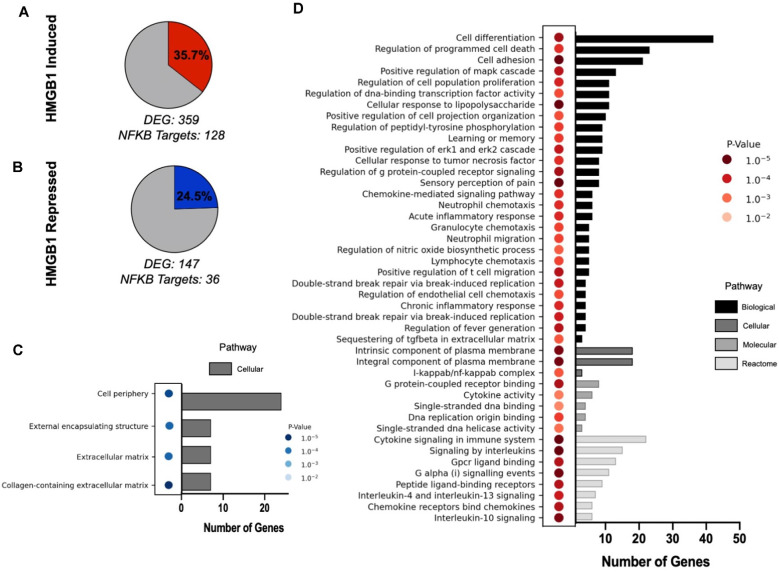
Gene ontology enrichment analysis of HMGB1 regulated NFκB gene pathways. Analysis of HMGB1 regulated transcriptional responses in OPCs identified differentially expressed NFκB target genes which reflected both induced **(A)** and repressed **(B)** patterns of gene expression. **(C)** GO analysis of repressed cellular processes in OPCs treated with HMGB1 as defined by NFκB-regulated transcriptional targets. No pathways in this analysis other than those listed had significant differences from control. **(D)** GO analysis of processes induced by HMGB1 identified biological pathways which emphasized cellular periphery as a major influence of HMGB1 on OPC activity. These data confirm the pathophysiological actions of HMGB1 *in vitro* and *in vivo* and provide a template for understanding of how HMGB1 potently affects OPC differentiation potential. P values depicted in **(C** and **D)** are indicated by color and the number of genes in each pathway category are indicated by a bar graph on the X axis for each panel.

Lastly, to interrogate the downstream effects of HMGB1 on OPC behavior, RNAseq datasets we generated previously (Nicaise et al., 2019) were used to identify HMGB1 regulated genes. We analyzed the differentially expressed genes (DEGs) which exhibited upregulation in the presence of HMGB1, and also downregulation in HMGB1 inhibited conditions, and cross-referenced DEG lists against lists of known NFκB regulated genes (Rouillard et al., 2016). This analysis of transcriptional datasets determined that NFκB target genes represented 35.7% of all significantly upregulated DEGs and also 24.5% of all downregulated DEGs in OPCs. Gene ontology analysis revealed that these NFκB regulated DEGs represented processes of cellular proliferation and differentiation that were consistent with HMGB1-induced impairment of OPC differentiation demonstrated both *in vivo* (Figure 1) and in our previous study (Nicaise et al., 2019). Together, these data strongly implicate NFκB-regulated transcription in impaired OPC-mediated remyelination and as a pathway directly regulated by HMGB1 (Figure 6).

## Discussion

Previous work in our lab has shown that HMGB1 was preferentially expressed by progenitor cells in progressive forms of MS and that this was coincident with these progenitors displaying a cellular senescent phenotype. This led to the hypothesis that elevated HMGB1 in progressive MS may be both an indicator and mediator of chronic demyelination. To further test the possibility that HMGB1 can negatively impact OPC-mediated remyelination, results of this study have shown that a single injection of HMGB1 given at the time of the LPC lesion is sufficient to significantly impair OPC differentiation and thus attenuated the degree of remyelination. However, this failure is not due to a lack of OPCs, as we saw comparable levels of OPCs in the non-HMGB1 and HMGB1 lesions. This observation that OPCs persist in lesions but fail to differentiate has been widely observed and documented in MS (Skaper, 2019). Herein, we have tested this hypothesis and our results show that HMGB1 can negatively impact remyelination *in vivo*, and we have identified TLR-NFκB signaling as a possible signaling mechanism in OPCs that mediates the effects of extracellular HMGB1 *in vitro*.

Since TLRs are known receptors for HMGB1, we had also tested the possibility that TLR-signaling may contribute to the effects of HMGB1 on OPCs. HMGB1 is not the first putative ligand reported for TLR2 as a means of affecting OLs. Indeed, TLR2 has previously been implicated as a receptor for hyaluronan and has been shown to block OPC maturation and remyelination (Sloane et al., 2010). The high molecular weight form of hyaluronan that is secreted by astrocytes has been shown to accumulate in chronically demyelinated lesions in both MS patients and mice with experimental autoimmune encephalomyelitis (EAE), and has also been shown to inhibit remyelination in LPC lesions (Back et al., 2005). It is an interesting possibility that these two significantly different molecules may be acting in similar ways on the same pathway to similar effect. It is important to qualify that although HMGB1 is recognized as a ligand for TLRs, inhibition of TLR2 by itself is known to promote OPC differentiation (Sloane et al., 2010). Thus, our findings cannot conclusively establish that TLR2 is the only receptor for HMGB1 because there remains the possibility that inhibiting TLR2 may have offset the effects of HMGB1 in a manner unrelated to direct ligand-receptor interaction. From this perspective HMGB1 and regulation of TLR2 signaling on OPCs may act as competing physiological antagonists in the regulation of OPC fate.

**Figure 7:**
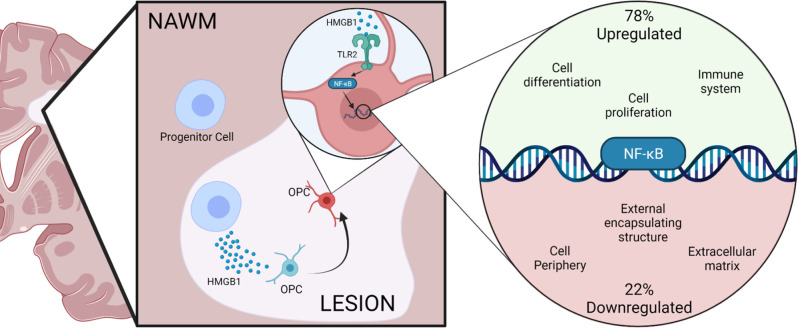
Hypothesized model of HMGB1 inhibitory activity on OPCs in the MS demyelinated lesion environment. Graphical representation of a demyelinated lesion in the MS brain and how expression of HMGB1 negatively impacts the remyelinating potential of OPCs. The stepwise progression from the MS brain (right) to the demyelinated lesion environment (middle panel) where HMGB1 is expressed at higher concentrations, to the intracellular response of OPCs (right panel) depicting a transcriptional influence of HMGB1 on NFκB gene expression regulation. Results from this study implicate TLR2-mediated receptor activation of NFκB gene target pathways as putative negative regulators of HMGB1 in the MS brain lesion environment. Created with BioRender.

Our data indicate that extracellular HMGB1 may act *via* TLRs expressed on OPCs to induce activation of NFκB signaling. This finding is consistent with previous work by others on the expression of TLR2 by OPCs and for a negative role for TLR2 activation on OPC differentiation potential (Sloane et al., 2010; Wasko et al., 2019). Interestingly, our work also pointed to a significant influence of HMGB1 on NFκB signaling, both as indicated by the rapid nuclear translocation of NFκB in HMGB1 treated OPCs and in terms of gene ontology analysis of pathways and transcriptional targets in OPCs regulated by HMGB1. Herein we report that extracellular HMGB1, which is also a factor found abundantly in the inflammatory milieu of the demyelinated lesion in the MS brain, is a potential inducer of NFκB signaling resulting in perturbed OPC remyelinating potential (Figure 7). Our *in vitro* studies which are corroborated by *in vivo* findings in the LPC lesion model, are in contrast to prior *in vivo* work implicating IFNγ as a driver of NFκB activity in OL (Stone et al., 2017). In the previous study, it was reported that blocking NFκB activation in OLs using cell-specific expression of IκBαΔN resulted in OL death and a hypomyelination phenotype during murine development (Stone et al., 2017), suggesting a positive function for NFκB signaling in promoting myelination. Whereas based on our findings, we consider NFκB signaling *via* TLR2 activation by HMGB1 to be an impediment to OL differentiation and remyelination. Indeed, our analysis of transcriptional changes in OPCs that are regulated by HMGB1 support these findings that blocking HMGB1 results in activation of gene pathways associated with myelination and OL maturation and these data provide a new perspective on regulatory pathways governing the fate potential of OPCs.

HMGB1 has been shown to activate NFκB downstream signaling through TLR2, TLR4, TLR9, and RAGE in several disease contexts (Lohani and Rajeswari, 2016). In diabetic neuropathy, HMGB1 increases NFκB activity (Alomar et al., 2021), while in diabetic retinopathy, HMGB1 has been shown to upregulate NFκB specifically by inhibiting the activity of IKB-α (Liang et al., 2020). IKB-α is a protein responsible for NFκB sequestration in the cytoplasm, where it is unable to perform its transcriptional functions (Liu et al., 2017). In cuprizone studies, drugs found to increase IκB levels were protective (Khaledi et al., 2021), while in developmental studies of the inflammatory molecule S100B, delays in maturation of OPCs were observed concordant to NFκB activation, albeit through the RAGE pathway (Santos et al., 2018).

The present findings and our past work point to a disease-relevant role for extracellular HMGB1 as a factor contributing to remyelination failure in MS. At present, it is unclear if the extracellular (secreted) form of HMGB1 is derived from a separate pool of HMGB1 in progenitor cells, or if the stressful conditions of MS cause nuclear HMGB1 to be modified and exported to the extracellular environment. Since it is known that HMGB1 has nuclear localization and export signals (Bonaldi et al., 2003) that facilitate its movement between the cytoplasm and the nucleus, it is plausible that erroneous signaling could contribute to the redirection of HMGB1 to the extracellular space. Curiously, HMGB1 has no secretory signal peptide, and is not known to be secreted through the Golgi apparatus. Yet, HMGB1 is released, at least by monocytes, after concentrations of cytosolic HMGB1 lead to translocation into secretory lysosomes (Bonaldi et al., 2003). Understanding these fundamental properties of intracellular management of HMGB1 protein could provide important insights into how to affect the release of HMGB1 from progenitor cells in the context of disease. On that point, we know that HMGB1 is expressed and produced by progenitor cells in the MS brain (Nicaise et al., 2019; Absinta et al., 2021) but we do not yet know why progenitor cells in the MS brain seem to acquire a cellular senescent phenotype related to excessive expression and production of HMGB1. Future studies may explore salient questions regarding identifying the critical signals that trigger generation of HMGB1 by a senescent cell in this disease, and how intracellular HMGB1 protein is managed/modified for extracellular release.

The focus of our study was the role of extracellular HMGB1 within demyelinated lesions within the MS brain. While we employed a reductionist approach to refine our understanding of how HMGB1 acts on primary OLs in culture, we recognize that there are other physiological activities attributed to how extracellular HMGB1 may impact the brain that were not examined in this study. For instance, HMGB1 has also been implicated in the breakdown of the BBB (Zhang et al., 2011; Nishibori et al., 2020), an integral component of MS pathophysiology (Balasa et al., 2021) and studies in MS patients corroborate this; serum levels of HMGB1 in MS patients are significantly higher than healthy controls (Bucova et al., 2020). Additionally, serum HMGB1 levels are also elevated in EAE. Blocking extracellular HMGB1 significantly lessens disease severity by attenuating *T* cell extravasation into the CNS (Robinson et al., 2013). Interestingly, HMGB1 is also a well-known mediator of inflammation (Gorgulho et al., 2019; Wang et al., 2019; Ciprandi et al., 2020; Liu et al., 2020; Nishibori et al., 2020; Yang et al., 2021). We had analyzed the degree of activated microglia/macrophages in HMGB1-LPC and control-LPC lesioned animals as a means to discern whether the impaired recovery in the HMGB1-treated animals was also reflective of an ongoing, active lesion. Our analysis from 10 dpl suggest that at this late timepoint the inflammatory response did not differ in between LPC-treated groups. It is important to point out that since this study did not characterize earlier post-lesion timepoints, we cannot exclude the possibility that HMGB1 may have had an effect on microglial responses within the lesion environment prior to our analysis. Nevertheless, the consequence of HMGB1 administration by 10 dpl suggests that microglia do not remain activated when significantly impaired remyelination was noted (Figure 7). We also recognize that the role of microglia in demyelination and MS is complex, potentially playing both beneficial and harmful roles (Voet et al., 2019). In particular, in this LPC model, proinflammatory microglia have been found to be robustly active and present in the earlier stages post-lesion (Baydyuk et al., 2019), and a transition from a pro-inflammatory (iNOS+ TNFα+ CCL2+) to a more regenerative phenotype (Arg-1+ CD206+ IGF-1+) has been associated with initiation of remyelination in the LPC model (Lloyd et al., 2019). Additional study will be required to test whether HMGB1 impairs remyelination through direct actions on OPCs, or engages microglial cells, or both, *in vivo*. When considered together, our findings in this study are distinct but complementary to these previous studies and serve to highlight that extracellular HMGB1 likely contributes to manifold pathological changes associated with demyelination and autoimmunity related to complex, multi-system diseases like MS.

Multiple sclerosis is not the only instance where extracellular HMGB1 may negatively impact white matter. In animal models of stroke, extracellular HMGB1 has been found to stimulate inflammation, albeit through TLR4 signaling (Yang et al., 2011) instead of TLR2, as we showed here. Patients with cerebral and myocardial ischemia have also been found to have elevated levels of HMGB1 in their blood serum (Goldstein et al., 2006), much in the same way MS patients have elevated blood serum levels of HMGB1 (Bucova et al., 2020). HMGB1 has also been implicated in the periphery, with its release from neurons linked to localized inflammation in animal models of nerve injury and arthritis (Yang et al., 2021). Furthermore, we have studied HMGB1 in the context of MS on account of its excessive expression and production of HMGB1 from progenitor cells which exhibit a cellular senescent phenotype (Davalos et al., 2013; Nicaise et al., 2019). Yet, our findings that HMGB1 can directly impair OL maturation may also have implications for Alzheimer’s disease and frontotemporal dementia, where HMGB1 released from astrocytes has been reported to promote senescence in addition to neuropathology (Gaikwad et al., 2021). In these cases, perturbation of mitochondrial function may contribute to the development of a cellular senescent state that is also characterized by elevated HMGB1 expression and extracellular release (Wiley et al., 2016). If HMGB1 is also promoting senescence in the MS brain, there is the potential for a positive feedback loop which could exacerbate the disease condition by having the dual consequence of promoting inflammation while blocking remyelination.

Present treatments for MS are immunomodulatory in nature, suppressing inflammation and inhibiting immune infiltration into the CNS, however, this does not fully protect patients from demyelination and eventual axonal loss (Brück et al., 2013). We propose that through advancing our understanding on the potential mechanism(s) by which HMGB1 functions in the context of limiting CNS remyelination, we may exploit this knowledge as a novel means to stimulate endogenous remyelination in the MS brain.

## Data Availability Statement

The datasets presented in this study can be found in online repositories. The names of the repository/repositories and accession number(s) can be found below: BioProject accession no. PRJNA524718.

## Ethics Statement

The animal study was reviewed and approved by Institutional Animal Care and Use Committees from UConn Health and Georgetown University.

## Author Contributions

Experimental design was conceived by MR and SC. *In vitro* studies were performed by MR and PS. *In vivo* studies were conceived by SC and JKH. *In vivo* studies were performed and analyzed by JH and HK. RNAseq analyses were performed by PS and MR. Manuscript was written by MR and SC with editorial contributions from all authors. All authors contributed to the article and approved the submitted version.

## Conflict of Interest

The authors declare that the research was conducted in the absence of any commercial or financial relationships that could be construed as a potential conflict of interest.

## Publisher’s Note

All claims expressed in this article are solely those of the authors and do not necessarily represent those of their affiliated organizations, or those of the publisher, the editors and the reviewers. Any product that may be evaluated in this article, or claim that may be made by its manufacturer, is not guaranteed or endorsed by the publisher.

## Footnotes

^1^ http://pantherdb.org/

^2^ http://www.geneontology.org/

## References

[B1] Absinta M. Maric D. Gharagozloo M. Garton T. Smith M. D. Jin J.. (2021). A lymphocyte-microglia-astrocyte axis in chronic active multiple sclerosis. Nature 597, 709–714. 10.1038/s41586-021-03892-7 34497421 PMC8719282

[B2] Alomar S. Y. Gheit R. E. A. E. Enan E. T. El-Bayoumi K. S. Shoaeir M. Z. Elkazaz A. Y.. (2021). Novel mechanism for memantine in attenuating diabetic neuropathic pain in mice via downregulating the spinal HMGB1/TRL4/NF-kB inflammatory axis. Pharmaceuticals (Basel) 14:307. 10.3390/ph14040307 33915770 PMC8065430

[B3] Andersson U. Wang H. Palmblad K. Aveberger A. C. Bloom O. Erlandsson-Harris H.. (2000). High mobility group 1 protein (HMG-1) stimulates proinflammatory cytokine synthesis in human monocytes. J. Exp. Med. 192, 565–570. 10.1084/jem.192.4.565 10952726 PMC2193240

[B4] Back S. A. Tuohy T. M. F. Chen H. Wallingford N. Craig A. Struve J.. (2005). Hyaluronan accumulates in demyelinated lesions and inhibits oligodendrocyte progenitor maturation. Nat. Med. 11, 966–972. 10.1038/nm1279 16086023

[B5] Balasa R. Barcutean L. Mosora O. Manu D. (2021). Reviewing the significance of blood-brain barrier disruption in multiple sclerosis pathology and treatment. Int. J. Mol. Sci. 22:8370. 10.3390/ijms22168370 34445097 PMC8395058

[B6] Baydyuk M. Cha D. S. Hu J. Yamazaki R. Miller E. M. Smith V. N.. (2019). Tracking the evolution of CNS remyelinating lesion in mice with neutral red dye. Proc. Natl. Acad. Sci. U S A 116, 14290–14299. 10.1073/pnas.1819343116 31235582 PMC6628798

[B8] Bonaldi T. Talamo F. Scaffidi P. Ferrera D. Porto A. Bachi A.. (2003). Monocytic cells hyperacetylate chromatin protein HMGB1 to redirect it towards secretion. EMBO J. 22, 5551–5560. 10.1093/emboj/cdg516 14532127 PMC213771

[B9] Bonetti B. Stegagno C. Cannella B. Rizzuto N. Moretto G. Raine C. S. (1999). Activation of NF-κB and c-*jun* transcription factors in multiple sclerosis lesions. Implications for oligodendrocyte pathology. Am. J. Pathol. 155, 1433–1438. 10.1016/s0002-9440(10)65456-9 10550297 PMC1866971

[B10] Brück W. Gold R. Lund B. T. Oreja-Guevara C. Prat A. Spencer C. M.. (2013). Therapeutic decisions in multiple sclerosis: moving beyond efficacy. JAMA Neurol. 70, 1315–1324. 10.1001/jamaneurol.2013.3510 23921521 PMC4106803

[B11] Bucova M. Majernikova B. Durmanova V. Cudrakova D. Gmitterova K. Lisa I.. (2020). HMGB1 as a potential new marker of disease activity in patients with multiple sclerosis. Neurol. Sci. 41, 599–604. 10.1007/s10072-019-04136-3 31728855

[B12] Chang A. Tourtellotte W. W. Rudick R. Trapp B. D. (2002). Premyelinating oligodendrocytes in chronic lesions of multiple sclerosis. N. Engl. J. Med. 346, 165–173. 10.1056/NEJMoa010994 11796850

[B13] Ciprandi G. Bellussi L. M. Passali G. C. Damiani V. Passali D. (2020). HMGB1 in nasal inflammatory diseases: a reappraisal 30 years after its discovery. Expert Rev. Clin. Immunol. 16, 457–463. 10.1080/1744666X.2020.1752668 32252560

[B14] Davalos A. R. Kawahara M. Malhotra G. K. Schaum N. Huang J. Ved U.. (2013). p53-dependent release of Alarmin HMGB1 is a central mediator of senescent phenotypes. J. Cell Biol. 201, 613–629. 10.1083/jcb.201206006 23649808 PMC3653366

[B15] Ellerman J. E. Brown C. K. De Vera M. Zeh H. J. Billiar T. Rubartelli A.. (2007). Masquerader: high mobility group box-1 and cancer. Clin. Cancer Res. 13, 2836–2848. 10.1158/1078-0432.CCR-06-1953 17504981

[B16] Faissner S. Gold R. (2019). Progressive multiple sclerosis: latest therapeutic developments and future directions. Ther. Adv. Neurol. Disord. 12:1756286419878323. 10.1177/1756286419878323 31598138 PMC6764045

[B17] Fancy S. P. Zhao C. Franklin R. J. (2004). Increased expression of Nkx2.2 and Olig2 identifies reactive oligodendrocyte progenitor cells responding to demyelination in the adult CNS. Mol. Cell. Neurosci. 27, 247–254. 10.1016/j.mcn.2004.06.015 15519240

[B18] Gaikwad S. Puangmalai N. Bittar A. Montalbano M. Garcia S. Mcallen S.. (2021). Tau oligomer induced HMGB1 release contributes to cellular senescence and neuropathology linked to Alzheimer’s disease and frontotemporal dementia. Cell Rep. 36:109419. 10.1016/j.celrep.2021.109419 34289368 PMC8341760

[B19] Goldstein R. S. Gallowitsch-Puerta M. Yang L. Rosas-Ballina M. Huston J. M. Czura C. J.. (2006). Elevated high-mobility group box 1 levels in patients with cerebral and myocardial ischemia. Shock 25, 571–574. 10.1097/01.shk.0000209540.99176.72 16721263

[B20] Gorgulho C. M. Romagnoli G. G. Bharthi R. Lotze M. T. (2019). Johnny on the spot-chronic inflammation is driven by HMGB1. Front. Immunol. 10:1561. 10.3389/fimmu.2019.01561 31379812 PMC6660267

[B21] Gudi V. Gingele S. Skripuletz T. Stangel M. (2014). Glial response during cuprizone-induced de- and remyelination in the CNS: lessons learned. Front. Cell. Neurosci. 8:73. 10.3389/fncel.2014.00073 24659953 PMC3952085

[B22] Hoarau J. J. Krejbich-Trotot P. Jaffar-Bandjee M. C. Das T. Thon-Hon G. V. Kumar S.. (2011). Activation and control of CNS innate immune responses in health and diseases: a balancing act finely tuned by neuroimmune regulators (NIReg). CNS Neurol. Disord. Drug Targets 10, 25–43. 10.2174/187152711794488601 21143144

[B23] Hori O. Brett J. Slattery T. Cao R. Zhang J. Chen J. X.. (1995). The receptor for advanced glycation end products (RAGE) is a cellular binding site for amphoterin. Mediation of neurite outgrowth and co-expression of rage and amphoterin in the developing nervous system. J. Biol. Chem. 270, 25752–25761. 10.1074/jbc.270.43.25752 7592757

[B24] Khaledi E. Noori T. Mohammadi-Farani A. Sureda A. Dehpour A. R. Yousefi-Manesh H.. (2021). Trifluoperazine reduces cuprizone-induced demyelination via targeting Nrf2 and IKB in mice. Eur. J. Pharmacol. 909:174432. 10.1016/j.ejphar.2021.174432 34416238

[B25] Lian Y. J. Gong H. Wu T. Y. Su W. J. Zhang Y. Yang Y. Y.. (2017). Ds-HMGB1 and fr-HMGB induce depressive behavior through neuroinflammation in contrast to nonoxid-HMGB1. Brain Behav. Immun. 59, 322–332. 10.1016/j.bbi.2016.09.017 27647532

[B26] Liang W. J. Yang H. W. Liu H. N. Q32an W. Chen X. L. (2020). HMGB1 upregulates NF-kB by inhibiting IKB-α and associates with diabetic retinopathy. Life Sci. 241:117146. 10.1016/j.lfs.2019.117146 31816325

[B27] Liu T. Son M. Diamond B. (2020). HMGB1 in systemic lupus erythematosus. Front. Immunol. 11:1057. 10.3389/fimmu.2020.01057 32536928 PMC7267015

[B28] Liu T. Zhang L. Joo D. Sun S. C. (2017). NF-κB signaling in inflammation. Signal Transduct. Targeted Ther. 2:17023. 10.1038/sigtrans.2017.23 29158945 PMC5661633

[B29] Lloyd A. F. Davies C. L. Holloway R. K. Labrak Y. Ireland G. Carradori D.. (2019). Central nervous system regeneration is driven by microglia necroptosis and repopulation. Nat. Neurosci. 22, 1046–1052. 10.1038/s41593-019-0418-z 31182869 PMC6597360

[B30] Lohani N. Rajeswari M. R. (2016). Dichotomous life of DNA binding high mobility group box1 protein in human health and disease. Curr. Protein Pept. Sci. 17, 762–775. 10.2174/1389203717666160226145217 26916160

[B31] Mi H. Muruganujan A. Thomas P. D. (2013). PANTHER in 2013: modeling the evolution of gene function and other gene attributes, in the context of phylogenetic trees. Nucleic Acids Res. 41, D377–D386. 10.1093/nar/gks1118 23193289 PMC3531194

[B32] Moore C. S. Milner R. Nishiyama A. Frausto R. F. Serwanski D. R. Pagarigan R. R.. (2011). Astrocytic tissue inhibitor of metalloproteinase-1 (TIMP-1) promotes oligodendrocyte differentiation and enhances CNS myelination. J. Neurosci. 31, 6247–6254. 10.1523/JNEUROSCI.5474-10.2011 21508247 PMC3090636

[B33] Mutukula N. Man Z. Takahashi Y. Iniesta Martinez F. Morales M. Carreon-Guarnizo E.. (2021). Generation of RRMS and PPMS specific iPSCs as a platform for modeling multiple sclerosis. Stem Cell Res. 53:102319. 10.1016/j.scr.2021.102319 33894548

[B34] Nicaise A. M. Banda E. Guzzo R. M. Russomanno K. Castro-Borrero W. Willis C. M.. (2017). iPS-derived neural progenitor cells from PPMS patients reveal defect in myelin injury response. Exp. Neurol. 288, 114–121. 10.1016/j.expneurol.2016.11.012 27865736

[B35] Nicaise A. M. Wagstaff L. J. Willis C. M. Paisie C. Chandok H. Robson P.. (2019). Cellular senescence in progenitor cells contributes to diminished remyelination potential in progressive multiple sclerosis. Proc. Natl. Acad. Sci. U S A 116, 9030–9039. 10.1073/pnas.1818348116 30910981 PMC6500153

[B36] Nishibori M. Wang D. Ousaka D. Wake H. (2020). High mobility group Box-1 and blood-brain barrier disruption. Cells 9:2650. 10.3390/cells9122650 33321691 PMC7764171

[B37] O’Connor K. A. Hansen M. K. Rachal Pugh C. Deak M. M. Biedenkapp J. C. Milligan E. D.. (2003). Further characterization of high mobility group box 1 (HMGB1) as a proinflammatory cytokine: central nervous system effects. Cytokine 24, 254–265. 10.1016/j.cyto.2003.08.001 14609567

[B38] Park J. S. Svetkauskaite D. He Q. Kim J. Y. Strassheim D. Ishizaka A.. (2004). Involvement of toll-like receptors 2 and 4 in cellular activation by high mobility group box 1 protein. J. Biol. Chem. 279, 7370–7377. 10.1074/jbc.M306793200 14660645

[B40] Psachoulia K. Chamberlain K. A. Heo D. Davis S. E. Paskus J. D. Nanescu S. E.. (2016). IL4I1 augments CNS remyelination and axonal protection by modulating T cell driven inflammation. Brain 139, 3121–3136. 10.1093/brain/aww254 27797811 PMC5382940

[B41] Robinson A. P. Caldis M. W. Harp C. T. Goings G. E. Miller S. D. (2013). High-mobility group box 1 protein (HMGB1) neutralization ameliorates experimental autoimmune encephalomyelitis. J. Autoimmun. 43, 32–43. 10.1016/j.jaut.2013.02.005 23514872 PMC3672339

[B42] Rouillard A. D. Gundersen G. W. Fernandez N. F. Wang Z. Monteiro C. D. Mcdermott M. G.. (2016). The harmonizome: a collection of processed datasets gathered to serve and mine knowledge about genes and proteins. Database (Oxford) 2016:baw100. 10.1093/database/baw100 27374120 PMC4930834

[B43] Santos G. Barateiro A. Gomes C. M. Brites D. Fernandes A. (2018). Impaired oligodendrogenesis and myelination by elevated S100B levels during neurodevelopment. Neuropharmacology 129, 69–83. 10.1016/j.neuropharm.2017.11.002 29126910

[B44] Skaper S. D. (2019). Oligodendrocyte precursor cells as a therapeutic target for demyelinating diseases. Prog. Brain Res. 245, 119–144. 10.1016/bs.pbr.2019.03.013 30961866

[B45] Sloane J. A. Batt C. Ma Y. Harris Z. M. Trapp B. Vartanian T. (2010). Hyaluronan blocks oligodendrocyte progenitor maturation and remyelination through TLR2. Proc. Natl. Acad. Sci. U S A 107, 11555–11560. 10.1073/pnas.1006496107 20534434 PMC2895128

[B47] Stone S. Jamison S. Yue Y. Durose W. Schmidt-Ullrich R. Lin W. (2017). NF-κB activation protects oligodendrocytes against inflammation. J. Neurosci. 37, 9332–9344. 10.1523/JNEUROSCI.1608-17.2017 28842413 PMC5607472

[B49] Voet S. Prinz M. Van Loo G. (2019). Microglia in central nervous system inflammation and multiple sclerosis pathology. Trends Mol. Med. 25, 112–123. 10.1016/j.molmed.2018.11.005 30578090

[B50] Wang H. Bloom O. Zhang M. Vishnubhakat J. M. Ombrellino M. Che J.. (1999). HMG-1 as a late mediator of endotoxin lethality in mice. Science 285, 248–251. 10.1126/science.285.5425.248 10398600

[B51] Wang M. Gauthier A. Daley L. Dial K. Wu J. Woo J.. (2019). The role of HMGB1, a nuclear damage-associated molecular pattern molecule, in the pathogenesis of lung diseases. Antioxid. Redox Signal. 31, 954–993. 10.1089/ars.2019.7818 31184204 PMC6765066

[B52] Wasko N. J. Kulak M. H. Paul D. Nicaise A. M. Yeung S. T. Nichols F. C.. (2019). Systemic TLR2 tolerance enhances central nervous system remyelination. J. Neuroinflammation 16:158. 10.1186/s12974-019-1540-2 31351476 PMC6660683

[B53] Watanabe H. Son M. (2021). The immune tolerance role of the HMGB1-RAGE axis. Cells 10:564. 10.3390/cells10030564 33807604 PMC8001022

[B54] Wiley C. D. Velarde M. C. Lecot P. Liu S. Sarnoski E. A. Freund A.. (2016). Mitochondrial Dysfunction induces senescence with a distinct secretory phenotype. Cell Metab. 23, 303–314. 10.1016/j.cmet.2015.11.011 26686024 PMC4749409

[B55] Wolswijk G. (1998). Chronic stage multiple sclerosis lesions contain a relatively quiescent population of oligodendrocyte precursor cells. J. Neurosci. 18, 601–609. 10.1523/JNEUROSCI.18-02-00601.1998 9425002 PMC6792542

[B56] Wolswijk G. (2002). Oligodendrocyte precursor cells in the demyelinated multiple sclerosis spinal cord. Brain 125, 338–349. 10.1093/brain/awf031 11844734

[B58] Yang H. Andersson U. Brines M. (2021). Neurons are a primary driver of inflammation *via* release of HMGB1. Cells 10:2791. 10.3390/cells10102791 34685772 PMC8535016

[B59] Yang Q. W. Lu F. L. Zhou Y. Wang L. Zhong Q. Lin S.. (2011). HMBG1 mediates ischemia-reperfusion injury by TRIF-adaptor independent Toll-like receptor 4 signaling. J. Cereb. Blood Flow Metab. 31, 593–605. 10.1038/jcbfm.2010.129 20700129 PMC3049514

[B57] Yang H. Tracey K. J. (2010). Targeting HMGB1 in inflammation. Biochim. Biophys. Acta 1799, 149–156. 10.1016/j.bbagrm.2009.11.019 19948257 PMC4533842

[B60] Zhang J. Takahashi H. K. Liu K. Wake H. Liu R. Maruo T.. (2011). Anti-high mobility group box-1 monoclonal antibody protects the blood-brain barrier from ischemia-induced disruption in rats. Stroke 42, 1420–1428. 10.1161/STROKEAHA.110.598334 21474801

